# Chemistry in Life and in Death

**Published:** 1885-08

**Authors:** George Watt


ARTICLE III.

### CHEMISTRY IN LIFE AND IN DEATH.

BY GEORGE WATT.

[Read before the Xenia Academy of Medicine ]

Chemistry is so boundless in its bearings, so infinite in its reachings, so constant and unremitting in its efforts, and so varied in its facts and affinities, that no human mind can grasp it in any of these various relations, nor can finite minds conceive the wide range of its possible inquiry, research or action. All will agree that its function is coextensive with matter; but as a science it is not pent up by material barriers. Extend your thought along the pathway of the material universe till you have reached, not only the farthest star or satellite, but till that thought, hitherto a wanderer, rests on the outermost atom of creation, and still, because the containing is more extended than the contained, there must be space beyond Now, whether or not this space is empty is known to the Infinite One,—to the possessor of all knowledge; but the only science that can investigate and report as to the presence or absence of matter therein, is called chemistry, the science that tyros propose to teach in a college course of a few weeks or months.

To gain something of an idea of the busy nature, the industry, the prying meddlesomeness of chemistry, it is best to confine our observations, for a time, to the action and influence of but a single element, as a view of the whole range would overtax our vision.

Because it is the most abundant, and perhaps the most observable, oxygen may be taken as a fit specimen to illustrate chemical action and energy.

And where shall we begin our observations? for where shall it be found? Or rather, where is it not? Most certainly it is not omnipresent; but if we ascend to the top of the highest mountain, it welcomes us in the refreshing breeze, or corrodes like a canker, in the character of ozone. Dig down into the deepest mines, or explore the most minute recesses of the secret cavern, and our presence fails even to surprise it. The oceans are its reservoirs, the clouds and the storms are its messengers, the earth is its treasure-house, and the rocks its safe-deposits. It is about us, in us, of us. If we walk, or if we work, we gain the force essential to the effort, by this wonderful agent, oft a tyrant, but now a servant, obedient to our will, combining with carbo-hydrogen compounds, which must be constituent materials of our physical bodies, and in the complex process, we find the chemical power of this element changed into vital force, and this with the mechanical force necessary to the accomplishment of the acts performed. It follows, as a matter of course, that the tissues oxidized to generate this force are devitalized, and thus we see the chemistry of death to the individual tissues, while it is the chemistry of life to the body corporeal.

This element, oxygen, gets into our bodies in two ways. It is an essential constituent of most, if not all parts of living tissues. And although it is built into our bodies in obedience to the vital processes and functions, it is ever true to chemistry,—to the laws of affinity; for no matter how many sciences are concerned in our make-up, there is no warfare among them. The oxygen that becomes identified as a component of living bodies is taken in with and appropriated from the food. That taken in by respiration can never be so assimilated, but is for purposes entirely different. The oxygen taken in by breathing is stored within (or at least among) the red corpuscles, not for the building up, but for the destruction of tissues. This oxygen is used to generate, or liberate force, which may be chemical, vital, and mechanical, in turn, on the principle of correlation.

And another thought is here necessary to a clear understanding of the processes concerned. We often find oxygen an irritating, corroding agent, yet it is a leading constituent of our balmy atmosphere which fans us with its gentlest zephyrs, "Where the Wind of the West breathes its softest sigh."

It is often as docile as the tamed lion that fondles the hand that feeds him; and again it is as furious as the same lion aroused in his wrath by a taste of blood. Nothing can seem more tame than either when quiescent; but aroused, one becomes the terror of the arena, and the other the fiercest and most destructive element of the material world. Think of the gentle breezes of June at the hour when Aurora gilds the eastern sky, and you have this element altogether amiable and pacific. Think again of a condition in which by correlation of forces, heat becomes affinity, and this voracious element must have Chicago for breakfast and Boston for lunch, and at the close of Time's day will take the whole earth for his supper. And how is this all explained?

We can advance at least a step in the direction of light by calling to our aid the principle of allotropy. Some chemical agents possess at least two distinct chemical states; and these are sometimes so very unlike each other that the identity of the agent is doubted. And this is all the more likely to occur when the substance is not obvious to our ordinary observation. If memory serves, an effort was made in this Academy to illustrate what is meant by allotropy, by considering iron in its active and passive states. Be this as it may, the illustration is a good one, and will bear a second use.

It is well known that when iron is introduced into a somewhat concentrated watery solution of nitric acid, it rapidly decomposes both the acid and the water. This is true of iron in its ordinary state, in which it is chemically active. The iron decomposes the fluids at its own expense. It is rapidly oxidized, and as atom after atom of its oxide is formed, it combines with equivalent of the remaining nitric acid, and nitrate of iron is the result. But when the iron is rendered passive before entering the acid solution, no chemical action occurs. Iron may be rendered passive by several processes; but a convenient one for those who wish to try the experiment is the following: Before immersing the iron, place it in contact with platinum, and introduce both metals in such way that the platinum first comes in contact with the acid solution. After the immersion, the platinum may be withdrawn, and the iron remains passive.

That oxygen has its allotropic conditions is evident from even slight considerations. Were it active when stored in the red corpuscles, it could not fail to burn the phosphorized fats found there, as scarcely anything is more combustible. Again, oxygen may be prepared by one of the ordinary methods, such as by the decomposition of potassium chlorate, and a strip of iron may be suspended in it, almost indefinitely, without showing signs of rust. In this state it may be rendered active by passing sparks of electricity through it; and in its active state it is called ozone.

In being set free from any of its combinations, oxygen escapes in its active state, in this obeying a general principle; for it is found by experiment that chemical reagents act with increased energy under such circumstances. They are often spoken of as being in a nascent state, when they have this increased affinity as a result of being just set free, or being just formed by combination, in the case of compounds, for it is not necessary that the agent be elementary in order to such manifestation.

It has been mentioned that the passage of electricity changes passive, or quiescent oxygen to active; and as there is always more or less electric disturbance in the atmosphere, it can never be free from active oxygen, or ozone. It follows, therefore, that oxidizable substances exposed to atmospheric influence are more or less oxidized. The oxygen is always present, and always a portion of it is ready for action. When, from any cause, electric disturbance is much increased, the proportion of ozone in the atmosphere becomes so great that the lining membrane of the air passages is badly irritated; and while some persons are more readily and to a greater extent disturbed in this way than others, and epidemic catarrh, usually called a "bad cold," prevails. Under the same circumstances the mechanic and the artisan find it difficult to prevent the rusting of their iron and steel instruments and tools.

If true, as we believe, and have already intimated, that the affinity resulting in oxidation within the system is, by correlation, changed into vital force, it seems advisable to now give the basis of voluntary effort of both mind and body. The oxygen is stored in the corpuscle in its passive state. While it remains in this condition, the corpuscle can carry it with impunity. It seems almost certain that the effort of will power can change it from the passive to the active state. Thus changed it acts on the tissue it touches, kills it, or at least a portion of its molecules, and this affinity is correlated into vital force which results in voluntary effort. How the will renders it active, and especially how it renders the right portion active, so that the proper molecules are oxidized, is beyond the range of our present knowledge; yet it is only one more illustration of the influence of mind over matter, analogous to the principle that "He spake, and it was done." And after all, we know as well how the will renders oxygen active as how electric sparks accomplish the same end. That is, we know nothing about either.

While the system is in proper health—all the organs and functions normal—the red corpuscles are able to receive and carry a due supply of passive oxygen. And it has been ascertained by careful experiment that the supply intended to originate, or rather liberate force for voluntary effort, of mind or body, is received and stored away during sleep. And this is the principal explanation of why we must sleep. As long as the will can so control the corpuscles that none of the stored oxygen becomes active except in obedience to the will, the individual is, or can be calm and steady. But when, from any cause, such control is not complete, involuntary motions, twitchings, etc., occur. An extreme illustration of this condition is found in *delirium tremens.*

When from exhaustion or other cause the will has lost control of the stored oxygen, if the supply is still abundant, there is great irritability or restlessness, while considerable physical strength remains; but when the supply is nearly exhausted, extreme debility accompanies the irritability. Various medicines are used for the purpose of restoring this lost control, among them coffee, tea, tobacco, opium, and even alcohol, the latter giving at best but very temporary relief. It is claimed, however, that alcohol gives indirect relief in such cases, by furnishing fuel for the straying oxygen. If this is true, and it seems reasonable, the oxidation of it may result in saving tissue from similar destruction by the merciless element.

By way of condensation of thoughts already expressed in a somewhat scattered arrangement, let us bear in mind that in the formation of tissues and structures of the body, oxygen plays an important part, that it is an essential and an abundant constituent of our bodies, present everywhere, as it claimed by some physiologists, in quantities sufficient to oxidize or burn up all the other elements found in association with it. In the formation of tissues and structures of living bodies, whether animal or vegetable, it exists in combinations which satiate its affinities, and it is therefore quiescent. As long as any tissue is alive and normal, it remains in this condition, and seems to have no disposition to disturb any vital process or structure. Whatever life force or vitality may be, it seems to afford even highly oxidizable materials the ability to resist the action of oxygen, at least in its passive state, as has been already referred to in reference to the phosphorized matter in the red corpuscles. Or, perhaps, it would be more philosophic to speak of vitality being able ordinarily to hold the oxygen to its passive condition. Most, if not all the text-books speak of vitality being one of the circumstances that modify affinity.

And further, in this condensation, let it be remembered that the circulating fluid, the blood is saturated with the same element, much of it passive, yet a portion of it as ozone, while other portions are in combination with carbon, hydrogen, phosphorus, sodium, potassium, etc.

And now, with the condition in life, how, and what in death? With vitality gone, the structures, lately so full of life, are masses of inert matter, subservient to the laws of the inorganic kingdom, and each element, or compound radical, is obliged to look out for itself, amid the many—the almost innumerable affinities awakened by the shock of death. In order to conceive how long it will take oxygen to assert its power over the dead tissues, and to begin its destructive work of decomposition, think how long it will take an electric spark, after the striking point has been reached, to pass through an atmosphere of oxygen, thereby rendering a portion of it active. In other words, the change is instantaneous, and as no part of the body is free from contact with oxygen, it follows that oxidation must begin as soon as vitality has ceased. No time is lost in the correlation of forces, for though matter may be quiescent, force can not. Think for a moment, about the possibility of gravitation being suspended.

The statement above, in reference to instantaneous action after the cessation of vital force, is not strong enough, for it often occurs that vitality becomes so enfeebled that affinity overpowers it and oxidation takes place before death. Accordingly we often see putrefaction while life is still retained.

This view of the case may suggest the still disputed question as to the precedence of chemical action and micro-organisms, in putrefaction. The oxygen is always there, and ready to act, and can not be prevented from thus acting, but by a change in the laws of affinity, which are laws of the Unchangeable God, and it is difficult to imagine that micro-organic germs can make better time, get their work in earlier, or show more efficient action, however much they may assist in the process as secondary agencies.

Not only is the body built originally, and also continuously nourished in strict obedience to chemical laws; but every morbid change and every diseased action are caused by and conducted in accordance with these same laws. We can not even think till a molecule of the gray matter of the brain has been oxidized as a burnt sacrifice on the altar of life. Not even the great tempter can turn our thoughts into vicious channels except by producing a chemical change in a portion of brain substance.

And on the other hand remedial agents can not arrest disease or restore the ailing body, without producing chemical change of texture and tissue. Some talk of dynamic medicines, or rather medicines which act dynamically; but so far, on soliciting an explanation of the terms used, we have found them simply veils for the concealment of ignorance. But even if we recognize a cure by dynamication, we have only admitted that force, as well as matter may be remedial, which has never been denied; but force can so act only by causing chemical change; or at least it can not act without producing such change.

Like matter, force is indestructible. One form of it may be changed into another, as light into heat, and that into electricity, and that into mechanical force, etc.; but in these, and similar correlations, nothing is lost. These occurring in connection with matter, however, must always result in chemical changes therein.

Bearing in mind the principle that force can not be inert, but must be active, we can understand some of the phenomena pertaining to chemical changes accompanying, or resulting from death. The elements and compound radicals, no longer controlled by affinity as modified by vitality, are, to an extent, liberated, and each seeks new companionship in obedience to affinity unmodified by vital force. On this principal much of the nitrogen in the system combines with hydrogen, while sulphur, and also phosphorous unite, in large proportions, with the element. The first of these compounds is alkali, and it further combines with the other two, which are acids, as well as with carbonic acid, which is also liberated abundantly from dead and putrefying bodies. And nothing in Nature is more self-evident than the fact that the force known as affinity, in its control of chemical reagents, is fully able to dispose of, and return to its kindred dust, any organic body deprived of life, without the aid of micro-organisms, maggots, or vultures. Yet it does not follow that it must reject the aid of these agents, in the disposition of the dead; but it does follow that neither germs, "nor any other creature," can be able to exclude chemical action in the process. When we bear in mind that the oxygen stored in the corpuscles is held passive by vital force, and that it is rendered active by death, we must recognize the immediate commencement of oxidation. The free electricity in the overcharged cloud will not likely await the departure of the cattle before striking the isolated tree in the pasture field, nor will affinity wait for the production of germs, and the commencement of their work, before starting active oxygen at its destructive processes. One force is as likely to wait as another. —*Ohio State Journal of Dental Science.*

